# The Commencement Exercises

**Published:** 1888-03

**Authors:** 


### THE COMMENCEMENT EXERCISES

of the Southern Medical College, of Atlanta, Ga., took place at DeGive's Opera House on the night of the 29th of February.

As the RECORD is ready for the press, we can only sketch briefly a few of the interesting portions of what took place, and will reserve a part for a future issue.

The occasion was one of unusual interest. The exercises were opened with a short and appropriate prayer by the Rev. John Jones, D. D.

Professor Nicolson read the following report:

*To the President and Board of Trustees:*

At the close of the Ninth Session of the Southern Medical College we are able to report a continuous advance in all respects, and increased facilities for instruction in all departments, and still greater thoroughness in the course of instruction. Since the close of the last session there has been added to the institution a dental department, from which you will have a separate report. In the medical department there has been in attendance during the session of 1887–'88 ninety-two students, forty of whom are in the senior class. Of these we present to you thirty-two as entitled, after thorough examination, to the degree of "Doctor of Medicine."

WM. PERRIN NICOLSON, Dean.

Dr. T. S. Powell, President of the Faculty, conferred the degree of "M. D." upon the following graduates:

CLASS I—J. F. Beck, Ga.; B. B. Bell, Ga.; J. M. Bosworth, Ga.; J. D. Bradley, Ga.

CLASS II—G. M. Burnett, Ga.; C. D. Coker, Ga.; A. B. Eaton, Tenn.; S. J. Gay, Ala.

CLASS III—G. W. Hill, Jr., S. C.; Romeo Hicks, N. C.; V. H. Hicks, Ga., C. H. Holland, Ga.; M. D. Jamerson, Ga.

CLASS IV—David Lindley, Tenn.; C. L. McCaul, N. C.; M. M. McGehee, Ga.; A. P. McNeil, S. C.; D. B. Moore, Ga.

CLASS V—L. W. Pitchford, Ga.; J. J. Poore, Ga.; J. B. Sanders, Ga.; J. R. Sewell, Ga.; J. M. Spence, Ga.

CLASS VI—J. N. Statum, Ala.; A. R. Stephens, Ala.; J. W. Thomas, La.; W. J. Tipton, Ala.; B. M. Trentham, Ga.

CLASS VII—J. E. Tucker, W. J. Westbrook, J. F. Williams, Moses Yarbrough.

Dr. D. S. Carpenter, as Dean of the Dental Department, made a fine report. Although the first term, and only four months since it was determined upon, there were twenty-seven students, of whom six had passed satisfactory examinations as candidates for graduation.

The following are the graduates in dentistry, to-wit:

Geo. C. Branse, of Tenn.; J. A. Wills, of Ga.; J. M. Reese, of Ga.; A. J. Boss, of Ga.; John A. Pepper, of Tenn.; Benno Wichert, Germany.

The faculty in the Dental, as in the Medical, Department of this school is of a high order.

PRIZES.—In the Medical Department prizes were confered on the following gentlemen, to wit:

First Honor—By the Faculty—B. B. Bell.

Second Honor—By the Faculty—A. P. McNiel.

Obstetrics—By Professor T. S. Powell—J. D. Bradley.

Surgery—By Professor J. McF. Gaston—W. J. Tipton.

Physiology—By Professor R. C. Word—A. R. Stephens.

Anatomy—By Professor W. P. Nicolson—C. H. Holland.

Materia Medica—By Professor G. G. Roy—J. N. Spence, Jr.

Practice—By Professor W. D. Bizzell—S. J. Gay.

Eye, Ear and Throat—By Professor A. G. Hobbs—G. W. Hill.

Chemistry—By Professor Burns—C. H. Holland.

Kiser Thesis Prize—A. B. Eaton.

Smith & Bradfield Prize—J. B. Sanders.

This last mentioned prize of $25 worth of drugs was by that large and reliable drug house of Smith & Bradfield, in this city, at the corner of Whitehall and Mitchell streets, a fine establishment and most excellent gentlemen.

The address of A. B. Eaton, valedictorian, was a fine production, and well delivered.

So the address of J. A. Wills, valedictorian of the Dental Department, was a well written and creditable one.

The address of Colonel Nisbet, the special orator of the occasion, contained so many valuable suggestions to the profession and the public, that we refrain from comment, with a view to publishing the entire address in a future number of the RECORD.

The prizes in the Dental Department are as follows:

First Honor—By the Faculty, gold medal—to Benno Wichert.

Prize in Anatomy—Gold medal, by Professor Nicolson—to A. J. Boss.

Prize in Philosophy—Drug cabinet, by Professor Word—to J. A. Wills.

Prize in Oral Surgery and Materia Medica—Gold medal, by Professor Henley—to Jno. A. Pepper.

Prize in Chemistry and Metalurgy—By Professor Holland—to J. M. Reese.

Prize in Operative and Clinical Dentistry—Set of instruments, by Professor Crenshaw—to J. A. Wills.

Prize in Mechanical and Practical Dentistry—Oxyhydrogen blow-pipe, by Professor Thompson—to B. Wichert.

Prize in Pathology and Therapeutics—By Professsor Carpenter—to A. J. Boss.

Special Prize in Junior Class—A gold medal, by Holliday Brothers—to A. Branch.

Professor Powell's remarks to the graduates on conferring the degrees were replete with sound principles and good advice, and was characterized by his usual eloquence of language and beauty of style.

An unusual and interesting episode occurred in the concluding part of the exercises—being two beautiful designs, exquisitely decorated with flowers, having emblematical significance—the one a cross, presented by Dr. John W. Thomas, of New Orleans, a member of the graduating class, and whose wife is an eminent lady practitioner of medicine, the other a star and crescent from the ladies of the Robert Morris Chapter of the Eastern Star of New Orleans. presented as an honor to the Southern Medical College, and in recognition of the efforts, the energy, and the rapid rise of the institution.

The address of Dr. B. Holly, the accomplished Episcopal minister of St. Philip's church, in presenting these beautiful designs, was most elegant and appropriate, and was admirably responded to by Professor Powell, of the Faculty.

